# Review of online educational resources for medical physicists

**DOI:** 10.1120/jacmp.v14i6.4476

**Published:** 2013-11-04

**Authors:** Joann I. Prisciandaro

**Affiliations:** ^1^ Department of Radiation Oncology University of Michigan Ann Arbor MI

**Keywords:** medical physics, education, online resources

## Abstract

Medical physicists are often involved in the didactic training of graduate students, residents (both physics and physicians), and technologists. As part of continuing medical education, we are also involved in maintenance of certification projects to assist in the education of our peers. As such, it is imperative that we remain current concerning available educational resources. Medical physics journals offer book reviews, allowing us an opportunity to learn about newly published books in the field. A similar means of communication is not currently available for online educational resources. This information is conveyed through informal means. This review presents a summary of online resources available to the medical physics community that may be useful for educational purposes.

PACS number: 87.10.‐e

## I. INTRODUCTION

With the widespread availability of the Internet, many educators are turning to online tools to enhance the educational experience of their students. The Internet provides us with a means of accessing electronic information and resources. It has provided us with a new means of communication. However, caution must be exercised when using data from the Internet as Web pages are not peer reviewed and can give misleading and sometimes incorrect information. Nonetheless, there are many Web sites, especially those sponsored by professional organizations, which provide material suitable for instructional purposes.

This review presents a summary of known professional society Web sites and educational sites frequented by the author. In addition, a PubMed search was performed on the key words “medical physics education.” It identified several potentially interesting Web sites. A brief description of these sites and their content is provided below. This list is not comprehensive due to the ever‐changing and evolving nature of the Internet. An attempt to compile a similar list was initiated in 2005 by the American Association of Physicist in Medicine (AAPM) Task Group Number 115, Educator's Resource Guide, and resulted in a list of medical physics‐related books, electronic media, journal articles, and Web sites (personal communication with D. Peck, February 27, 2013). This information was not made available publically, but an updated and expanded summary of the online educational resources based on TG 115's work is presented in Table 1.

**Table 1:** List of relevant online educational sites based on the initial work of AAPM TG 115. Each of the hyperlinks is categorized by the educational content they contain

Professional Societies and Organizations										
			*Educational Categories*							
*Intended Audience*	*Author*	*Hyperlink*	*Physics/Radiologic Physics*	*Anatomy & Physiology*	*Health Physics*	*Radiation Biology*	*Diagnostic Imaging*	*Nuclear Medicine*	*Radiation Therapy*	*Misc*.
Physicist	American Association of Physicists in Medicine (AAPM)	http://www.aapm.org	X		X		X	X	X	
	AAPM Medical Physics Resource Page	http://www.aapm.Org/links/medphys/default.asp#lists	X		X		X	X	X	
Physicist, Physician	American Board of Radiology (ABR)	http://theabr.org								X
	ABR Foundation	http://www.abrfoundation.org					X		X	
Physicist, Physician	American College of Radiology (ACR)	http://www.acr.org			X		X	X	X	
	ACR Care in Point	http://3s.acr.org/cip/		X			X			
Physicist, Technologist	Association of Educators in Imaging and Radiological Sciences (AEIRS)	http://www.aeirs.org								X
Physicist	American Institute of Physics (AIP)	http://www.aip.org/education/	X							
General	American Institute of Ultrasound in Medicine (AIUM)	http://www.aium.org/				X				
General	American Nuclear Society (ANS)	http://www.ans.org	X							
Physician	Association of Program Directors in Radiology (APDR)	http://www.apdr.org/residents_and_fellows.cfm								X
Physicist, Physician	American Society for Therapeutic Radiology and Oncology (ASTRO)	http://www.astro.org				X			X	
Physicist	Canadian College of Physicists in Medicine (CCPM)	http://www.ccpm.ca	X		X		X	X	X	
Physicist	Canadian Organization of Medical Physicists (COMP)	http://www.medphys.ca/	X		X		X	X	X	
Physicist, Physician	Canadian Partnership for Quality Radiotherapy (CPQR)	http://www.cpqr.ca/tqc.htm1							X	
General	Conference of Radiation Control Program Directors (CRCPD)	http://www.crcpd.org								X
General	Digital Imaging and Communications in Medicine (DICOM)	http://medical.nema.org/								X
Physicist, Physician	European Society for Radiotherapy and Oncology (ESTRO)	http://www.estro.org	X			X			X	
	ESTRO Brachytherapy Handbook	http://www.estro‐education.org/publications/Documents/GEC ESTRO Handbook of Brachytherapy.html	X			X			X	
General	The Joint Commission	http://www.jointcommission.org/								X
Physicist	Health Physics	http://www.hps.org Society (HPS)			X					
Physician	Intersocietal Accreditation Commission (IAC)	http://www.intersocietal.org/echo					X			
General	Institute for Magnetic Resonance Safety, Education, and Research (IMRSER)	http://www.imrser.org/default.asp					X			
General	Institute of Physics (IOP)	http://physicsweb.org/resources/home	X							
Physicist	Institute of Physics and Engineering in Medicine (IPEM)	www.ipem.ac.uk	X				X	X	X	
General	International Society for Magnetic Resonance in Medicine (ISMRM)	http://www.ismrm.org/resources/mr‐sites/	X	X			X			
Physicist, Physician	Radiation Therapy Oncology Group(RTOG)	www.rtog.org/Home.aspx		X					X	
	RTOG Contouring Atlases	http://www.rtog.org/CoreLab/ContouringAtlases.aspx		X						
General	Radiation Research Society (RADRES)	http://www.radres.org								X
Physician	Radiology Education Foundation	http://www.refindia.net/rlinks/reviewedlinks/portals.htm		X			X			
Physician	Radiological Society of North America (RSNA)	http://rsna.org/	X				X			
	RSNA Science & Education	http://rsna.org/education/physics.cfm	X				X			
	RSNA Education Offerings	http://www.rsna.org/Education_Offerings.aspx	X				X			
General	RSNA/ACR	http://www.radiologyinfo.org/		X			X	X	X	
General	Society of Nuclear Medicine and Molecular Imaging	http://www.snm.org/		X			X	X		
Physicist	International Society for Optics and Photonics	http://www.spie.org					X			
*National and International Agencies*										
			*Educational Categories*							
*Intended Audience*	*Author*	*Hyperlink*	*General Radiologic Physics*	*Anatomy & Physiology*	*Health Physics*	*Radiation Biology*	*Diagnostic Imaging*	*Nuclear Medicine*	*Radiation Therapy*	*Misc*.
General	Food and Drug Administration (FDA)	http://www.fda.gov	X		X					
General	US Government Information (FedWorld)	http://fedworld.ntis.gov	X		X					
Physicist	International Atomic Energy Agency (IAEA)	http://www.iaea.org	X		X	X	X	X	X	
	IAEA Division of Human Health	http://www‐naweb.iaea.org/NAHLI/index.html			X	X	X	X	X	
	IAEA Radiation Oncology Physics Handbook	http://www‐naweb.iaea.org/nahu/DMRP/syllabus.html	X		X				X	
	IAEA Slides of Radiation Oncology Physics Handbook	http://www‐naweb.iaea.org/nahu/DMRP/slides.html	X		X				X	
	IAEA Radiation Biology Handbook	http://www‐pub.iaea.org/MTCD/publications/PDF/TCS‐42_web.pdf				X				
	IAEA Distance Learning Course in Radiation Oncology	http://www.iaea.org/Publications/Training/Aso/register.html							X	
	IAEA Radiation Protection of Patients (RPOP)	https://rpop.iaea.org/RPOP/RPoP/Content/index.htm			X		X	X	X	
	RPOP Publications	https://rpop.iaea.org/RPOP/RPoP/Content/AdditionalResources/Publications/index.htm			X		X	X	X	
	RPOP Free material	https://rpop.iaea.org/RPOP/RPoP/Content/AdditionalResources/Training/1TrainingMaterial/index.htm			X		X	X	X	
Physicist	International Commission on Radiological Protection (ICRP)	http://www.icrp.org			X	X	X	X	X	
Physicist	National Institute of Standards and Technology	http://www.nist.gov/pml/data/xraycoef/	X							
General	Nuclear Regulatory Commission	http://www.nrc.gov			X			X	X	
General	UK National Radiological Protection Board (NRPB)	http://www.nrpb.org/			X					
General	National Technical Information Service (NTIS)	http://www.ntis.gov	X		X					

*University and College Websites*											
				*Educational Categories*							
*Intended Audience*	*Author*	*Hyperlink*	*Additional Information*	*Physics/Radiologic Physics*	*Anatomy & Physiology*	*Health Physics*	*Radiation Biology*	*Diagnostic Imaging*	*Nuclear Medicine*	*Radiation Therapy*	*Misc*.
Physician	Hall, E.C.	http://www.columbia.edu/~ejh1/web‐rad‐train/review.html	Radiologic review material	X			X				
Physicist, Physician	Barclay, A.	http://www.emory.edu/CRL/abb	Medical imaging resource page	X	X			X	X		
Physicist	European Medical Radiation Learning Development (EMERALD)	http://www.emerald2.eu/emerald_index.html	Training modules in medical radiation physics	X				X	X	X	
	http://www.emitel2.eu/emitwwwsql/index‐login.aspx	Medical physics encyclopedia and multilingual dictionary									X
		http://emerald2.eu/mep/e‐book11/ETCBOOK2011_ebook_s.pdf	Medical physics training book	X				X	X	X	
Physicist, Physician	Harvard University	http://www.med.harvard.edu/AANLIB/home.html	Whole Brain Atlas		X						
Physicist	Idaho State University Health Physics Society (ISUHPS)	http://www.physics.isu.edu/radinf/index.html	Radiation information network	X		X					
Physician	Mallinckrodt Inst. Radiology Nuc Med Faculty	http://gamma.wustl.edu/home.html	Teaching Files	X	X			X	X		
Physicist, Physician	Mallinckrodt Inst. Radiology Teaching Files	http://www.rad.Washington.edu/anatomy/index.html	Anatomy of the skeleton		X						
Physicist	Oak Ridge Associated Universities (ORAU)/Oak Ridge National Laboratory (ORNL)	http://www.orau.org/ptp/infores.htm	Health physics resources	X		X					
Physicist, Physician	Homak, J.P.	http://www.cis.rit.edu/htbooks/mri	Basics of MRI	X	X			X			
Physicist	Redish, E.F.	http://www2.physics.umd.edu/~redish/Book/	Link to textbook *Teaching physics with the physics suite*	X							
General	University of North Carolina	http://www.med.unc.edu/radonc/pro/education	Computer Based Learning Modules for Clinical Dosimetry	X		X	X	X		X	
Physicist	Woo, M. and K.H. Ng	http://www.biij.org/rremp/Remote	Real‐Time Learning (RRTL)	X		X		X	X	X	
*Miscellaneous Websites*											
				*Educational Categories*							
*Intended Audience*	*Author*	*Hyperlink*	*Additional Information*	*Physics/Radiologic Physics*	*Anatomy & Physiology*	*Health Physics*	*Radiation Biology*	*Diagnostic Imaging*	*Nuclear Medicine*	*Radiation Therapy*	*Misc*.
Physician	Bergman, R.A.	http://www.anatomyatlases.org/	Anatomy Atlases		X						
Physician	Corporate	http://www.auntminnie.com	Discussion forum and news for radiologists		X			X	X		
Physicist, Physician	CHORUS site	http://chorus.rad.mew.edu	Collaborative Hypertext of Radiology		X			X			
Physicist, Physician	Medical Imageworks LLC	http://www.idoimaging.com/index.shtml_	Resource of free medical imaging software								X
Physician	Herring, W.	http://www.leamingradiology.com/	Radiology teaching files		X			X			
General	Medcalc	http://www.medcalc.com/	On‐line clinical calculators								X
Physician	MedPix	http://rad.uuhs.edu/medpix/medpix.html?mode=tf2#top	Radiological images		X			X			
Physicist, Physician	Pawlicki, T., S. Mutic, Brown, D., and P. Dunscombe	http://treatsafely.org	Resources to improve quality and safety in radiation medicine							X	X
	Pawlicki, T., S. Mutic, Brown, D., and P. Dunscombe	https://i.treatsafely.org	Resources to improve quality and safety in radiation therapy							X	X
Physician	Ballinger, R.	http://www.mritutor.org/	Radiology/MRI teaching files		X			X			
Physician	D'Alessandro, M.P.	http://www.pediatricradiology.com/	Discussion forum and educational resources		X			X			
Physicist, Physician	Radiation Dose Assessment Resource (RADAR)	http://www.doseinfo‐radar.com	Dose assessment resource			X			X		
Physician	D'Alessandro, M.P.	http://www.radiologyeducation.com/	General Radiology teaching files		X			X			
Physician	Lewis, E.	http://www.radiologyweb.com/	Discussion forum and educational resources		X			X			
Physicist	unknown	http://www.revisemri.com/	MRI educational material	X				X			
General	Ness‐Aiver, M.	http://www.simplyphyics.com/MAIN.HTM	MRI physics material	X							
General	Sprawls, P.	http://www.sprawls.org/ppmi2,/	Medical physics resource material	X		X		X			
	Sprawls, P.	http://www.sprawls.org/resources/	X		X		X			
General	1 Ultrarad	http://www.lultrarad.com	Ultrasound Imaging resources					X			
General	Medical Education	http://hub.webring.org/hub/mededrg	Collection of sites related to Medical education					X			
General	unknown	http://www.xray2000.co.uk/	Medical imaging educational resources		X			X			
Physician	Mclaughlin, P.	http://www.prostadoodle.com/	Prostate atlas		X						
General	unknown	http://www.radhelper.com/	Discussion forum		X			X			
Physicist, Physician	Ahmad, N.	http://www.radquiz.com/	Radiology Information X	X	X			X			
Physicist	Beavis, A.W., R. Phillips, and J.W. Ward	http://www.vertual.co.uk/	Virtual Environment for Radiotherapy Training (VERT)							X	

## MEDICAL PHYSICS EDUCATIONAL RESOURCES

### A. List servers

There are a number of official mailing lists that are geared specifically for medical physics. These include the following:

- DXIMGMEDPHYS ‐ Diagnostic imaging medical physicist list
- MEDPHYSUSA ‐ American medical physics mailing list
- MEDPHYS ‐ The Global medical physics mailing list
- ○ This mailing list is intended to be a forum of communication for the international medical physics community.^(1)^
- MEDPHYSBOARDPREPARATION ‐ Medical physics board preparation study group
- ○ This list server is intended to attract and assist medical physics trainees that are in the process of taking their medical physics board exams.^(2)^

Instructions on how to subscribe to the DXIMGMEDPHYS, MEDPHYSUSA, and MEDPHYS mailing lists are provided on the AAPM website, at www.aapm.org/links/medphys/. To join the MEDPHYSBOARDPREPARATION mailing list, one may perform a search of YAHOO! Groups, or visit health.groups.yahoo.com/group/medphysboardpreparation.

### B. Professional organizations and national/international agency Web sites

### B.1 American Association of Physicists in Medicine (AAPM)

The AAPM is a nonprofit organization dedicated to the scientific, educational, and professional advancement of physics in medicine.^(3)^ To achieve this end, the AAPM Web site has a host of resources available to its members. Links are available to journals such as *Medical Physics*, the *Journal of Applied Clinical Medical Physics*, and *Physics Today*. In addition, the AAPM has over one hundred reports summarizing the findings of specially‐convened task groups and working groups on clinical, scientific, professional, and educational topics available to download free to members and the public.

The AAPM's Virtual Library maintains copies of recorded presentations that were given at annual AAPM meetings and specialty medical physics meetings, as well as vendor presentations from AAPM corporate affiliates. The recordings include streaming video and/or audio of the presenter, slides, and/or audio transcriptions. The material contained is extensive and useful to educators, students, and community physicists.

For an additional fee, the On‐line Learning Center (OLC) is available for AAPM members to aid in their Maintenance of Certification (MOC) process. The OLC offers online educational presentations and quizzes, allowing users to obtain online SAMs credits.

The AAPM Educators Resource Guide provides curriculum guidance, online modules, and references to medical physicists involved in education. The guide is currently organized into six categories. The most developed is the Physics Education for Diagnostic Radiologists and Residents. This guide provides links to the AAPM/RSNA physics tutorials,^(4)^ slide show presentations, and online modules, such as Dr. Perry Sprawls' online resources for learning and teaching the physical principles of medical imaging.^(5)^

There are efforts underway to improve the online didactic content available on the AAPM Web site. Several subcommittees and task groups have been charged with this endeavor, including the Online Learning Services Subcommittee and Task Group Number 206, On‐line Didactic Content.

#### B.2 American Board of Radiology Foundation (ABRF)

The ABRF is an independent, nonprofit organization whose mission is to demonstrate, enhance, and continually improve accountability in the use of medical imaging and radiotherapy.^(^ ^6^ ^,^ ^7^ ^)^ To achieve this end, the ABRF has begun hosting annual summits and has worked to develop a professionalism series.

The intention of the annual summit is to address national health‐care challenges such as overutilization of medical imaging (2009), improving patient care through electronic communication in imaging (2010), and safe use in medical imaging (2012). A summary of the summit agendas and select presentations are available for free downloads on the ABRF Web site (www.abrfoundation.org).

The professionalism series resulted in the development of ten online ethics and professionalism modules. The modules were developed by a team of experts and the content has been peer reviewed. The development of these modules was financially supported by the ABRF, the Academy of Radiology Research (ARR), the AAPM, the American Board of Radiology (ABR), the American College of Radiology (ACR), the American Radium Society (ARS), the American Society for Radiation Oncology (ASTRO), and the Radiological Society of North America (RSNA), and the modules are available free to members of any of these organizations. The modules are self‐guided and include tests and practicums to allow users to assess their comprehension of the material.

#### B.3 American Society of Radiation Oncology (ASTRO)

ASTRO is an independently managed organization that is dedicated to improving the quality of patient care through education, clinical practice, research, and advocacy.^(8)^ Although the vast majority of its members are radiation oncology physicians, medical physicists make up approximately 17% of ASTRO's total membership (personal communication with ASTRO Membership Department, February 14, 2012). Consequently, the majority of the educational resources available on the ASTRO Web site are designed for physicians. However, there are still online educational resources that are relevant to medical physicists. These include Self‐Assessment Modules, webinars, and virtual meetings, which are available for a nominal fee.

#### B.4 European Society for Radiotherapy and Oncology (ESTRO)

ESTRO is an international organization dedicated to the advancement and support of education, research, and networking across all areas of radiation oncology.^(9)^ ESTRO's membership has an international and multidisciplinary flair, spanning five continents and including specialists involved in all aspects of the multimodality treatment of cancer. To support the educational needs of its members and the radiotherapy and oncology community, ESTRO has published a series of useful resources on its educational portal. This includes curricula, guidelines, publications, and e‐learning tools. There are two online publications that may be most interesting for medical physicists. The first is the *GEC‐ESTRO Handbook of Brachytherapy*.^(10)^ The handbook provides readers with a comprehensive summary of clinical presentations and brachytherapy techniques used to treat various anatomical sites by the most innovative European teams, as well as basic principles of physics, radiobiology, and imaging. The second is the ESTRO physics series. The series consists of ten booklets on an array of clinical medical physics topics such as *in vivo* dosimetry, quality assurance, monitor unit calculations, and intensity‐modulated radiation therapy.

To minimize travel and expense, ESTRO also offers a series of e‐learning resources. ESTRO's Application for Global Learning (EAGLE) provides video and audio material, presentations, text, and virtual classroom sessions. In addition, the Fellowship in Anatomic deLineation and CONtouring (FALCON) and the Tutorial for Image Guided External Radiotherapy (TIGER) provide presentations, lessons, and online courses to help guide and validate the contouring techniques of professionals such as physicians, physicists, and dosimetrists.

#### B.5 Health Physics Society (HPS)

HPS is a professional scientific organization that promotes excellence in the science and practice of radiation safety.^(11)^ The HPS issues a number of publications that are free to its members and available for a nominal fee to nonmembers. These include *Health Physics News, Health Physics Journal, Operational Radiation Safety*, conference proceedings, and position and policy papers. The HPS also has a number of complimentary topical articles available related to issues such as instrumentation, medical and dental patients, nonionizing radiation, and radiation effects.

#### B.6 International Atomic Energy Agency (IAEA)

The IAEA is an international organization that seeks to promote safe, secure, and peaceful use of nuclear technology.^(^ ^12^ ^,^ ^13^ ^)^ The IAEA's mission is guided by the interests and needs of its member states.^(12)^ Of particular interest to the medical community is the Division of Human Health within the Department of Nuclear Sciences and Applications. The objective of the Division of Human Health is to address the needs of member states related to the use of nuclear technology to prevent, diagnosis, and treat health‐related issues.^(14)^ To address these needs, the Division is further subdivided into four sections: (1) Nuclear Medicine, (2) Applied Radiation Biology and Radiotherapy, (3) Dosimetry and Medical Radiation Physics, and (4) Nutritional and Health‐Related Environmental Studies. The Web site of each section provides useful links, a frequently asked questions page, and free resources related to the medical application of nuclear technology. This includes a number of educational resources such as:

- Radiation Oncology Physics: a handbook for teachers and students^(15)^
- Radiation Oncology Physics Slides^(16)^
- Radiation Biology: a handbook for teachers and students^(17)^
- Distance learning course in radiation oncology for cancer treatment^(18)^

Another section of interest on the IAEA Web site is the Radiation Protection of Patients (RPOP) site.^(19)^ The intention of this Web site is to disseminate information to help health‐care professionals safely utilize radiation in medicine. A discussion on standards and prevention of errors is provided for professionals specializing in areas such as radiology, radiotherapy, nuclear medicine, interventional fluoroscopy, and interventional cardiology. Other publications such as safety guides, safety reports, technical documents, and radiological accidents are available for free downloads under the additional resource page.^(20)^ In addition, this site provides free training modules on the following topics:^(21)^

- Diagnostic and interventional radiology
- Radiotherapy
- Nuclear medicine
- Prevention of accidental exposure in radiotherapy
- Cardiology
- PET/CT
- Pediatric radiology
- Digital radiology

#### B.7 International Commission on Radiological Protection (ICRP)

The ICRP is an independent international organization that provides recommendations on the safe use of ionizing radiation.^(22)^ These recommendations are intended to provide assistance to the appropriate regulatory agencies of individual countries to develop radiological protection standards, legislation, guidelines, programs, and codes of practice.

The ICRP has published more than one hundred reports regarding radiation protection that are available electronically for a fee or free to subscribers of the Annals of the ICRP. In addition, this material may be downloaded either free or at a discounted rate to developing countries. The ICRP also has a number of summary recommendations, guides and explanatory notes, presentations summarizing various ICRP reports, and educational material available free to download from its Web site.

#### B.8 Radiological Society of North America (RSNA)

RSNA is a professional organization committed to excellence in patient care through education and research.^(23)^ To promote this mission, the RSNA Science & Education portal was developed. Within this portal, RSNA Education offerings includes links to:^(24)^

1. Online education
2. Resources from RSNA annual meeting
3. Ethics and professionalism modules
4. Resources from the Academy of Radiology Leadership and Management (ARLM)
5. Professionalism resources
6. RSNA/AAPM Physics modules
7. Educational offerings tutorial
8. Maintenance of Certification

Many of the resources available on this Web site may be downloaded or viewed free of charge.

#### B.9 Society of Nuclear Medicine and Molecular Imaging (SNMMI)

SNMMI is a nonprofit scientific and professional organization which promotes the use of nuclear medicine and molecular imaging.^(25)^ Its education page provides links to the SNMMI learning center, online continuing education lectures, and a molecular imaging resident webinar training series. For a nominal fee, the learning center will allow visitors access to online lectures and workshops, diagnostic CT and PET/CT cases, and Nuclear Medicine and PET study guides.

### C. Miscellaneous Web sites

#### C.1 EMERALD, EMIT, EMITEL

In 1995 following the European Conference on Post‐Graduate Education in Medical Radiation in Budapest, delegates from several European Union (EU) universities and hospitals engaged in a pilot project called European Medical Radiation Learning Development (EMERALD).^26^, ^27^, ^28^, ^29^ The goal of this project was to develop training material, such as curricula and e‐learning modules, to improve the training of medical physicists in the area of diagnostic radiology, nuclear medicine, and radiotherapy.^(26)^ Shortly after the development of the initial modules, a second EU‐sponsored project was initiated and titled European Medical Imaging Technology (EMIT). Similar to EMERALD, the EMIT project was initiated to develop medical physics training material but, in this case, focusing on diagnostic ultrasound and magnetic resonance imaging.^(26)^ Both projects continue to be developed and enhanced. At present, EMERALD II, a new and larger consortium has been organized. The training materials and workbooks are available online from the EMERALD II Web site, www.emerald2.eu/cd/Emerald2 ^(27)^ (personal communication with S. Tabolov, August 13, 2012).

The EMERALD II Web site also contains several other useful educational links, including EMITEL. The *European Medical Imaging Technology e‐Encyclopaedia for Lifelong Learning* (EMITEL) is a free medical physics electronic encyclopedia and multilingual dictionary.^1(30)^ The dictionary can be used to define and translate terms into anyone of 29 languages. The Medical Engineering and Physics (MEP) portal currently provides a link to two e‐books. The first e‐book, *Medical Radiation Physics from a European Perspective*, is the conference proceedings from the 1994 European Conference on Post‐Graduate Education in Medical Radiation, which describes the status of medical physics education in a number of European countries. The second e‐book, *Medical Physics and Engineering Education and Training Part I*, was published in 2011. This e‐book is a collection of papers from educational conferences summarizing the experience of medical physics education and training both within and outside of the EU, including the experience of educators in African, Asian, and South American countries. The two e‐books are available for free downloads online, and a third e‐book, a continuation of the 2011 publication, is planned for release in 2012–2013.^(31)^

#### C.2 Remote Real‐Time Education in Medical Physics (RREMP)

The RREMP project (formally known as Remote Real‐Time Learning (RRTL)) was initiated with the expressed interest of developing a means of providing medical physics didactic training to physicists living in small communities and/or remote regions of the world where access to formal, classroom‐based learning might be limited or nonexistent^35^, ^36^, ^37^ (personal communication with M. Woo, June 12, 2013). Through a collaborative effort between the Department of Medical Physics at the Toronto‐Sunnybrook Regional Cancer Centre and the Department of Radiology at the University of Malaysia, a pilot project was initiated and is on‐going. The objective of the RREMP project is to develop a simple, widely accessible, and cost‐effective method to allow real‐time interactive education and consultation via the Internet. This project has been piloted with a class of medical physics graduate students at the University of Malaysia, and the results are promising, thus giving credence to the viability and economic feasibility of real‐time interactive remote education.

#### C.3 Computer‐based learning modules for clinical dosimetry

A new computer‐based learning initiative is underway at the Department of Radiation Oncology at the University of North Carolina School of Medicine^(^ ^35^ ^,^ ^36^ ^)^ (personal communication with R.D. Adams, April 26, 2012, and with E.M. Zeman, April 24, 2012). A team headed by Dr. Robert Adams has developed a series of educational modules that would allow students to develop and hone practical, hands‐on skills using a treatment planning system (PLanUNC). The modules consist of didactic and interactive components, and are designed to allow students to apply their newly acquired knowledge in topics such as target delineation, dose calculations, electron dosimetry, treatment planning (beginning with two‐field plans), beam modifiers, and anatomic planning considerations^(35)^ (personal communication with E.M. Zeman, April 24, 2012). Short quizzes are given to the students pre‐ and post‐module completion to assess their level of comprehension and retention.^(35)^ The modules are currently geared to radiation therapy and dosimetry students; however, their utility for radiation oncology and medical physics residents will be evaluated at a later date. The Computer Based Learning Modules for Clinical Dosimetry is expected to be available online by the end of the summer of 2013.

#### C.4 Virtual Environment for Radiotherapy Training (VERT)

In response to a shortage of clinical resources and equipment time available for the training of staff and students in radiotherapy, a team of two computer scientists and one medical physicist from the University of Hull and the Princess Royal Hospital embarked on a research project in 2001 that culminated in a product known as Virtual Environment for Radiotherapy Training (VERT; Vertual, East Yorkshire, UK)^37^, ^38^, ^39^, ^40^, ^41^, ^42^, ^43^ (personal communication with J. Antons, J. Ward, and A. Beavis, April 26‐May 10, 2012). VERT is an immersive, life‐size, virtual environment that allows staff and students to enter a virtual linac suite and practice setting up a virtual patient and using radiotherapy equipment.^(^ ^37^ ^,^ ^38^ ^)^ The virtual linac modeled in VERT has all of the movement and the majority of the functionality of a true linac. Users may choose from anatomic datasets provided by the vendor or import anatomized images via a DICOM RT import tool to model a patient. In addition to providing a visualization of the radiotherapy treatment vault, VERT includes:^(37)^

- Anatomic views of the patient on the patient table, including an internal view of patient to investigate the relationship of isocenter with the planning target volume (PTV) and organs at risk (OARs)
- Visualization of the treatment beams
- Visualization of the radiation dose distribution (i.e., isodose surfaces)
- Collision detection
- Automated skin marking tools
- Tools to quantify setup errors

Recently, the scope of the VERT system has been expanded to include a range of medical physics equipment such as:^(42)^

- Scanning water phantom
- Solid water QA block/ion chamber
- Light/radiation coincidence phantom
- Laser alignment phantom
- Water based calibration phantom

VERT can be operated in demonstrator or virtual simulator mode, depending on the desired utility.^(37)^ In simulator mode, the 3D glasses worn by the user interfaces with the computer/projection system and tracks the position of the observer. As a result, the system creates an observer's eye view of the virtual linac suite and “allows the user to ‘walk around’ an object, viewing it from different aspects”.^(39)^

The VERT system consists of a 3D projector, screen, 3D glasses, high‐performance computer(s), audio system, and linac hand pendant(s) that controls the linac and treatment couch^(39)^ (personal communication with R.D. Adams, April 26, 2012). Although VERT can be tailored to accommodate the space available at a given facility, to truly appreciate the virtual experience, VERT should be projected at a life‐size scale^(39)^ (personal communication with J. Antons, J. Ward, and A. Beavis, April 26‐May 10, 2012).

At the time of writing, VERT has been installed in 81 facilities in 14 countries. These installations include 28 fully immersive VERT systems and 53 seminar‐style systems (personal communication with J. Antons, J. Ward, and A. Beavis, April 26‐May 10, 2012).

## V. CONCLUSIONS

The advent of the World Wide Web and multimedia technology has transformed how instructors and learners approach education.^(44)^ Not only has the Web provided us with a means of accessing instant online resources, it has also provided us with a new means of communicating and interacting. Online educational tools offer novel and compelling instructional resources. They provide instructors and learners with new educational venues.^(44)^ Furthermore, as technologies continue to evolve and become increasingly integrated into our culture and society, it is important that we, as educators, adapt and tailor our approach to education to better suit the needs of today's learners.^(44)^ This review was intended to provide a summary of useful online educational resources; however, the author is aware that the list of sites presented (see Table 2) is not comprehensive due to the evolving nature of the Internet. An attempt to address this shortcoming was initiated in 2005 by the American Association of Physicists in Medicine (AAPM) Task Group Number 115, Educator's Resource Guide (personal communication with D. Peck, February 27, 2013). However, this information was not made available publically. Discussions to revitalize this project have commenced within AAPM Subcommittee on Medical Physicists as Educators.

**Table 2:** List of hyperlinks to educational Web sites presented in the current review

*List Servers*	
*Intended Audience*	*Mailing List*	*Hyperlink or Contact to subscribe*
Physicist	DXIMGMEDPHYS	listserv@hermes.gwu.edu (Details for subscription available at http://www.aapm.org/links/medphys/.)
Physicist	MEDPHYSUSA	listserv@lists.wayne.edu or lists.wayne.edu/archives/medphysusa.html
Physicist	MEDPHYS	listserv@lists.wayne.edu or lists.wayne.edu/archives/medphys.html
Physicist	MEDPHYSBOARDPREPARATION	http://health.groups.yahoo.com/group/medphysboardpreparation/
*Professional Organizations and National/International Agency Web Sites*		
*Intended Audience*	*Organization/Agency*	*Hyperlink*
Physicist	American Association of Physicists in Medicine (AAPM)	http://www.aapm.org
Physicist, Physician	ABR Foundation	http://www.abrfoundation.org
Physicist, Physician	American Society for Therapeutic Radiology and Oncology (ASTRO)	http://www.astro.org
Physicist, Physician	European Society for Radiotherapy and Oncology (ESTRO)	http://www.estro.org
Physicist	Health Physics Society (HPS)	http://www.hps.org
Physicist	International Atomic Energy Agency (IAEA)	http://www.iaea.org
Physicist	International Commission on Radiological Protection (ICRP)	http://www.icrp.org
Physician	Radiological Society of North America (RSNA)	http://rsna.org/
General	Society of Nuclear Medicine and Molecular Imaging	http://www.snm.org/
*Miscellaneous Web Sites*		
*Intended Audience*	*Group/Title*	*Hyperlink*
Physicist	European Medical Radiation Learning Development (EMERALD)	http://www.emerald2.eu/emeraldindex.html http://emerald2.eu/cd/Emerald2/
Physicist	European Medical Imaging Technology e‐Encyclopaedia for Lifelong Learning (EMITEL)	http://www.emitel2.eu/emitwwwsql/index‐login.aspx
Physicist	Remote Real‐Time Education in Medical Physics (RREMP)	http://www.biij.org/rremp/
General	Computer Based Learning Modules for Clinical Dosimetry	http://www.med.unc.edu/radonc/pro/education
Physicist	Virtual Environment for Radiotherapy Training (VERT)	http://www.vertual.co.uk/

## ACKNOWLEDGMENTS

The author would like to thank Drs. Jay Burmeister and Donald Peck for their lively discussion and feedback related to the direction of the presented review project. Additionally, the author would like to thank Dr. Peck for his willingness to share the educator's resource guide initially developed by AAPM TG 115. The author would also like to acknowledge the contributions of Drs. Peter Dunscombe and Victor Montemayor to the listed content in Table 1.

## Supporting information

Supplementary Material

Supplementary Material
