# Supplementary material for: Review of online educational resources for medical physicists
Source: J Appl Clin Med Phys. 2013 Nov 4;14(6):368–87. doi: 10.1120/jacmp.v14i6.4476 (PMC5714624; doi:10.1120/jacmp.v14i6.4476)
Supplement: Supplementary file 1 — Supplementary Material [file ACM2-14-368-s001.docx]

***Review of Online Educational Resources for Medical Physicists***

**Joann I. Prisciandaro PhD^1^**

^1^Department of Radiation Oncology, University of Michigan, Ann Arbor, MI

Corresponding Author:

Joann I. Prisciandaro, Ph.D., DABR

University of Michigan Hospital and Health Systems

Department of Radiation Oncology

1500 East Medical Center Dr.

UH B2 C438, SPC 5010

Ann Arbor, MI 48109

734-936-4309 (office)

734-936-7859 (fax)

[joannp@med.umich.edu](mailto:joannp@med.umich.edu)

Running Title: Review of Online Educational Resources

The author has no financial disclosures or conflicts of interest.
