# Supplementary material for: Review of online educational resources for medical physicists
Source: J Appl Clin Med Phys. 2013 Nov 4;14(6):368–87. doi: 10.1120/jacmp.v14i6.4476 (PMC5714624; doi:10.1120/jacmp.v14i6.4476)
Supplement: Supplementary file 2 — Supplementary Material [file ACM2-14-368-s002.doc]

Review of Online Educational Resources for Medical Physicists

**Joann I. Prisciandaro, Ph.D.**

*Department of Radiation Oncology, University of Michigan, Ann Arbor, MI*

Corresponding author:

*Joann I. Prisciandaro, Ph.D.*

*University of Michigan Hospital and Health Systems*

*Department of Radiation Oncology*

*1500 East Medical Center Dr.*

*UH B2 C438, SPC 5010*

*Ann Arbor, MI 48109*

*Tel.: (734) 936 4309
Fax: (734) 936 7859*
Email: joannp@med.umich.edu

Submitted: March 31, 2013

Accepted: July 24, 2013

Running title: Review of Online Educational Resources

The author has no financial disclosures or conflicts of interest.

**Review of Online Educational Resources for Medical Physicists**

# Abstract:

Medical Physicists are often involved in the didactic training of graduate students, residents (both physics and physician), and technologists. As part of continuing medical education, we are also involved in maintenance of certification projects to assist in the education of our peers. As such, it is imperative that we remain current of the available educational resources available to us. Medical Physics journals offer Book Reviews, allowing us an opportunity to learn about newly published books in the field. A similar means of communication is not currently available for online educational resources. This information is conveyed through informal means. This review presents a summary of online resources available to the medical physics community that may be useful for educational purposes.

*Key words: Medical Physics, Education, Online Resources*

*PACS numbers: 87.10.-e*

# Introduction

With the widespread availability of the internet, many educators are turning toward online tools to enhance the educational experience of their students. The internet provides us with a means of accessing electronic information and resources. It has provided us with a new means of communication. However, caution must be exercised when utilizing data from the internet as web pages are not peer reviewed and can give misleading and sometimes, incorrect information. Nonetheless, there are many websites, especially those sponsored by professional organizations, which provide material suitable for instructional purposes.

This review presents a summary of known professional society websites and educational sites frequented by the author. In addition, a PubMed search was performed on the key words “medical physics education.” It identified several potentially interesting websites. A brief description of these sites and their content is provided below. This list is not comprehensive due to the ever-changing and evolving nature of the internet. An attempt to compile a similar list was initiated in 2005 by the American Association of Physicist in Medicine (AAPM) Task Group No. 115, Educator’s Resource Guide, and resulted in a list of medical physics related books, electronic media, journal articles, and websites . This information was not made available publically, but an updated and expanded summary of the online educational resources based on TG 115’s work is presented in Table 1.

# Medical Physics Educational Resources

## List servers

There are a number of official mailing lists that are geared specifically for medical physics. These include the following:

- DXIMGMEDPHYS – Diagnostic imaging medical physicist list
- MEDPHYSUSA – American medical physics mailing list
- MEDPHYS – The Global medical physics mailing list
  - This mailing list is intended to be a forum of communication for the international medical physics community .
- MEDPHYSBOARDPREPARATION – Medical physics board preparation study group
  - This list server is intended to attract and assist medical physics trainees that are in the process of taking their medical physics board exams .

Instructions on how to subscribe to the DXIMGMEDPHYS, MEDPHYSUSA, and MEDPHYS mailing lists are provided on the AAPM website, at [www.aapm.org/links/medphys/](http://www.aapm.org/links/medphys/). To join the MEDPHYSBOARDPREPARATION mailing list one may perform a search of YAHOO! Groups, or visit health.groups.yahoo.com/group/medphysboardpreparation.

## Professional Organizations and National/International Agency Websites

#### American Association of Physicists in Medicine (AAPM)

The AAPM is a non-profit organization dedicated to the scientific, educational, and professional advancement of physics in medicine . To achieve this end, the AAPM website has a host of resources available to its members. Links are available to journals such as Medical Physics, the Journal of Applied Clinical Medical Physics, and Physics Today. In addition, the AAPM has over one hundred reports summarizing the findings of specially-convened task groups and working groups on clinical, scientific, professional, and educational topics available to download free to members and the public.

The AAPM’s Virtual Library maintains copies of recorded presentations that were given at annual AAPM meetings and specialty medical physics meetings, as well as vendor presentations from AAPM corporate affiliates. The recordings include streaming video and/or audio of the presenter, slides, and/or audio transcriptions. The material contained is extensive and useful to educators, students and community physicists.

For an additional fee, the On-line Learning Center (OLC) is available for AAPM members to aid in their Maintenance of Certification (MOC) process. The OLC offers on-line educational presentations and quizzes, allowing users to obtain on-line SAMs credits.

The AAPM Educators Resource Guide provides curriculum guidance, on-line modules, and references to medical physicists involved in education. The guide is currently organized into six categories. The most developed is the Physics Education for Diagnostic Radiologists and Residents. This guide provides links to the AAPM/RSNA physics tutorials , slide show presentations, and on-line modules, such as Dr. Perry Sprawls’ online resources for learning and teaching the physical principles of medical imaging .

There are efforts underway to improve the on-line didactic content available on the AAPM website. Several subcommittees and task groups have been charged with this endeavor, including the Online Learning Services Subcommittee and Task Group No. 206, On-line didactic content.

#### American Board of Radiology Foundation (ABRF)

The ABRF is an independent, non-profit organization whose mission is to demonstrate, enhance, and continually improve accountability in the use of medical imaging and radiotherapy . To achieve this end, the ABRF has begun hosting annual summits and has worked to develop a professionalism series.

The intention of the annual summit is to address national healthcare challenges such as overutilization of medical imaging (2009), improving patient care through electronic communication in imaging (2010), and safe use in medical imaging (2012). A summary of the summit agendas and select presentations are available for free downloads on the ABRF website ([www.abrfoundation.org](http://www.abrfoundation.org/)).

The professionalism series resulted in the development of ten on-line ethics and professionalism modules. The modules were developed by a team of experts and the content has been peer reviewed. The development of these modules was financially supported by the ABRF, the Academy of Radiology Research (ARR), the AAPM, the American Board of Radiology (ABR), the American College of Radiology (ACR), the American Radium Society (ARS), the American Society for Radiation Oncology (ASTRO), and the Radiological Society of North America (RSNA), and are available free to members of any of these organizations. The modules are self-guided and include tests and practicums to allow users to assess their comprehension of the material.

#### American Society of Radiation Oncology (ASTRO)

ASTRO is an independently managed organization that is dedicated to improving the quality of patient care through education, clinical practice, research, and advocacy . Although the vast majority of its members are radiation oncology physicians, medical physicists make up approximately 17% of ASTRO’s total membership . Consequently, the majority of the educational resources available on the ASTRO website are designed for physicians. However, there are still on-line educational resources that are relevant to medical physicists. These include Self-Assessment Modules, webinars, and virtual meetings, which are available for a nominal fee.

#### European Society for Radiotherapy and Oncology (ESTRO)

ESTRO is an international organization dedicated to the advancement and support of education, research, and networking across all areas of radiation oncology . ESTRO’s membership has an international and multidisciplinary flair, spanning five continents and including specialists involved in all aspects of the multimodality treatment of cancer. To support the educational needs of their members and the radiotherapy and oncology community, ESTRO has published a series of useful resources on their educational portal. This includes curricula, guidelines, publications, and e-learning tools. There are two on-line publications that may be most interesting for medical physicists. The first is the GEC-ESTRO Handbook of Brachytherapy. The Handbook of Brachytherapy provides readers with a comprehensive summary of clinical presentations and brachytherapy techniques used to treat various anatomical sites by the most innovative European teams, basic principles of physics, radiobiology, and imaging. The second is the ESTRO physics series. The series consists of ten booklets on an array of clinical medical physics topics such as in vivo dosimetry, quality assurance, monitor unit calculations, and intensity-modulated radiation therapy.

To minimize travel and expense, ESTRO also offers a series of e-learning resources. ESTRO’s Application for Global Learning (EAGLE) provides video and audio material, presentations, text, and virtual classroom sessions. In addition, the Fellowship in Anatomic deLineation and CONtouring (FALCON) and the Tutorial for Image Guided External Radiotherapy (TIGER) provide presentations, lessons and on-line courses to help guide and validate the contouring techniques of professionals such as physicians, physicists, and dosimetrists.

#### Health Physics Society (HPS)

HPS is a professional scientific organization that promotes excellence in the science and practice of radiation safety . The HPS issues a number of publications that are free to its members and available for a nominal fee to nonmembers. These include Health Physics News, Health Physics Journal, Operational Radiation Safety, conference proceedings, and position and policy papers. The HPS also has a number of complimentary topical articles available related to issues such as instrumentation, medical and dental patients, nonionizing radiation, and radiation effects.

#### International Atomic Energy Agency (IAEA)

The IAEA is an international organization that seeks to promote safe, secure, and peaceful use of nuclear technology . The IAEA’s mission is guided by the interests and needs of its member states . Of particular interest to the medical community is the Division of Human Health within the Department of Nuclear Sciences and Applications. The objective of the Division of Human Health is to address the needs of member states related to the use of nuclear technology to prevent, diagnosis, and treat health-related issues . To address these needs, the Division is further subdivided into four sections: (1) Nuclear Medicine, (2) Applied Radiation Biology and Radiotherapy, (3) Dosimetry and Medical Radiation Physics, and (4) Nutritional and Health-Related Environmental Studies. Each section website provides useful links, a frequently asked questions page, and free resources related to the medical application of nuclear technology. This includes a number of educational resources such as:

- Radiation Oncology Physics: A handbook for teachers and students
- Radiation Oncology Physics Slides
- Radiation Biology: A handbook for teachers and students
- Distance learning course in radiation oncology for cancer treatment

Another section of interest on the IAEA website is the Radiation Protection of Patients (RPOP) website . The intention of this website is to disseminate information to help healthcare professionals safely utilize radiation in medicine. A discussion on standards and prevention of errors is provided for professionals specializing in areas such as radiology, radiotherapy, nuclear medicine, interventional fluoroscopy, and interventional cardiology. Other publications such as safety guides, safety reports, technical documents, and radiological accidents are available for free downloads under their additional resource page . In addition, this site provides free training modules on the following topics :

- Diagnostic and interventional radiology
- Radiotherapy
- Nuclear medicine
- Prevention of accidental exposure in radiotherapy
- Cardiology
- PET/CT
- Pediatric radiology
- Digital radiology

#### International Commission on Radiological Protection (ICRP)

The ICRP is an independent international organization that provides recommendations on the safe use of ionizing radiation . These recommendations are intended to provide assistance to the appropriate regulatory agencies of individual countries to develop radiological protection standards, legislation, guidelines, programs, and codes of practice.

The ICRP has published more than one hundred reports regarding radiation protection that are available electronically for a fee or free to subscribers of the Annals of the ICRP. In addition, this material may be downloaded either free or at a discounted rate to developing countries. The ICRP also has a number of summary recommendations, guides and explanatory notes, presentations summarizing various ICRP reports, and educational material available free to download from their website.

#### Radiological Society of North America (RSNA)

RSNA is a professional organization committed to excellence in patient care through education and research . To promote this mission, the RSNA Science & Education portal was developed. Within this portal, RSNA Education offerings includes links to :

1. Online education
2. Resources from RSNA annual meeting
3. Ethics and professionalism modules
4. Resources from the Academy of Radiology Leadership and Management (ARLM)
5. Professionalism resources
6. RSNA/AAPM Physics modules
7. Educational offerings tutorial
8. Maintenance of Certification

Many of the resources available on this website may be downloaded or viewed free of charge.

#### Society of Nuclear Medicine and Molecular Imaging (SNMMI)

SNMMI is a nonprofit scientific and professional organization which promotes the use of nuclear medicine and molecular imaging . Their education page provides links to the SNMMI learning center, online continuing education lectures, and a molecular imaging resident webinar training series. For a nominal fee, the learning center will allow visitors access to online lectures and workshops, diagnostic CT and PET/CT cases, and Nuclear Medicine and PET study guides.

## Miscellaneous Websites

#### EMERALD, EMIT, EMITEL

In 1995, following the European Conference on Post-Graduate Education in Medical Radiation in Budapest, delegates from several European Union (EU) universities and hospitals engaged in a pilot project called European Medical Radiation Learning Development (EMERALD) . The goal of this project was to develop training material, such as curricula and e-learning modules, to improve the training of medical physicists in the area of diagnostic radiology, nuclear medicine, and radiotherapy . Shortly after the development of the initial modules, a second EU sponsored project was initiated and titled European Medical Imaging Technology (EMIT). Similar to EMERALD, the EMIT project was initiated to develop medical physics training material, but in this case, focusing on diagnostic ultrasound and magnetic resonance imaging . Both projects continue to be developed and enhanced. At present, EMERALD II, a new and larger consortium has been organized. The training materials and workbooks are available on-line from the EMERALD II website, [www.emerald2.eu](http://www.emerald2.eu/)/cd/Emerald2 .

The EMERALD II website also contains several other useful educational links, including EMITEL. The European Medical Imaging Technology e-Encyclopaedia for Lifelong Learning (EMITEL) is a free medical physics electronic encyclopedia and multilingual dictionary . The dictionary can be used to define and translate terms into anyone of 29 languages. The Medical Engineering and Physics (MEP) portal currently provides a link to two e-books. The first e-book, “Medical Radiation Physics from a European Perspective,” is the conference proceedings from the 1994 European Conference on Post-Graduate Education in Medical Radiation, which describes the status of medical physics education in a number of European countries. The second e-book, “Medical Physics and Engineering Education and Training Part I” was published in 2011. This e-book is a collection of papers from educational conferences summarizing the experience of medical physics education and training both within and outside of the EU, including the experience of educators in African, Asian, and South American countries. The two e-books are available for free downloads on-line, and a third e-book, a continuation of the 2011 publication, is planned to be released in 2012 – 2013 .

#### Remote Real-Time Education in Medical Physics (RREMP)

The RREMP project (formally known as Remote Real-Time Learning (RRTL)) initiated with the expressed interest of developing a means of providing medical physics didactic training to physicists living in small communities and/or remote regions of the world, where access to formal, classroom-based learning might be limited or nonexistent . Through a collaborative effort between the Department of Medical Physics at the Toronto-Sunnybrook Regional Cancer Centre and the Department of Radiology at the University of Malaysia, a pilot project was initiated and is on-going. The objective of the RREMP project is to develop a simple, widely accessible, and cost-effective method to allow real-time interactive education and consultation via the internet. This project has been piloted with a class of medical physics graduate students at the University of Malaysia, and the results are promising; thus giving credence to the viability and economic feasibility of real-time interactive remote education.

#### Computer Based Learning Modules for Clinical Dosimetry

A new computer based learning initiative is underway at the Department of Radiation Oncology at the University of North Carolina School of Medicine . A team headed by Dr. Robert Adams has developed a series of educational modules that would allow students to develop and hone practical, hands-on skills using a treatment planning system (PLanUNC). The modules consist of didactic and interactive components, and are designed to allow students to apply their newly acquired knowledge in topics such as target delineation, dose calculations, electron dosimetry, treatment planning (beginning with 2 field plans), beam modifiers, and anatomic planning considerations . Short quizzes are given to the students pre and post module completion to assess their level of comprehension and retention . The modules are currently geared to radiation therapy and dosimetry students; however, their utility for radiation oncology and medical physics residents will be evaluated at a later date. The Computer Based Learning Modules for Clinical Dosimetry is expected to be available on-line by the end of the summer of 2013.

#### Virtual Environment for Radiotherapy Training

In response to a shortage of clinical resources and equipment time available for the training of staff and students in radiotherapy, a team of two computer scientists and one medical physicist from the University of Hull and the Princess Royal Hospital embarked on a research project in 2001 that culminated in a product known as Virtual Environment for Radiotherapy Training (VERT, copyright Vertual, East Yorkshire, UK) . VERT is an immersive, life-size, virtual environment that allows staff and students to enter a virtual linac suite and practice setting up a virtual patient and using radiotherapy equipment . The virtual linac modeled in VERT has all of the movement and the majority of the functionality of a true linac. Users may choose from anatomic datasets provided by the vendor or import anatomized images via a DICOM RT import tool to model a patient. In addition to providing a visualization of the radiotherapy treatment vault, VERT includes :

- Anatomic views of the patient on the patient table, including an internal view of patient to investigate the relationship of isocenter with the planning target volume (PTV) and organs at risk (OARs).
- Visualization of the treatment beams.
- Visualization of the radiation dose distribution (i.e. isodose surfaces).
- Collision detection.
- Automated skin marking tools.
- Tools to quantify setup errors.

Recently, the scope of the VERT system has been expanded to include a range of medical physics equipment such as a :

- “Scanning water phantom
- Solid water QA block/ion chamber
- Light/radiation coincidence phantom
- Laser alignment phantom
- Water based calibration phantom”

VERT can be operated in demonstrator or virtual simulator mode depending on the desired utility . In simulator mode, the 3D glasses worn by the user interfaces with the computer/projection system and tracks the position of the observer. As a result, the system creates an observer’s eye view of the virtual linac suite and “allows the user to ‘walk around’ an object, viewing it from different aspects.”

The VERT system consists of a 3D projector, screen, 3D glasses, high performance computer(s), audio system, and linac hand pendant(s) that controls the linac and treatment couch . Although VERT can be tailored to accommodate the space available at a given facility, to truly appreciate the virtual experience, VERT should be projected at a life-size scale .

At the time of writing, VERT has been installed in 81 facilities within 14 countries worldwide. These installations include 28 fully immersive VERT systems and 53 seminar style systems .

# Conclusions

The advent of the World Wide Web and multimedia technology has transformed how instructors and learners approach education . Not only has the web provided us with a means of accessing instant, on-line resources, it has also provided us with a new means of communicating and interacting. On-line educational tools offer novel and compelling instructional resources. They provide instructors and learners with new educational venues . Furthermore, as technologies continue to evolve and become increasingly integrated into our culture and society, it is important that we, as educators, adapt and tailor our approach to education to better suit the needs of today’s learners . This review was intended to provide a summary of useful online educational resources; however, the author is aware that the list of sites presented (see Table 2) is not comprehensive due to the evolving nature of the internet. An attempt to address this shortcoming was initiated in 2005 by the American Association of Physicists in Medicine (AAPM) Task Group No. 115, Educator’s Resource Guide . However, this information was not made available publically. Discussions to revitalize this project have commenced within AAPM Subcommittee on Medical Physicists as Educators.

**Acknowledgements**

The author would like to thank Drs. Jay Burmeister and Donald Peck for their lively discussion and feedback related to the direction of the presented review project. Additionally, the author would like to thank Dr. Peck for his willingness to share the educator’s resource guide initially developed by AAPM TG 115. The author would also like to acknowledge the contributions of Drs. Peter Dunscombe and Victor Montemayor to the listed content in Table 1.

# References

**Table Caption**

Table 1: List of relevant online educational sites based on the initial work of AAPM TG 115. Each of the hyperlinks is categorized by the educational content they contain.

Table 2: List of hyperlinks to educational websites presented in the current review.
